# Implementation of the Participatory Approach to increase supervisors’ self-efficacy in supporting employees at risk for sick leave; design of a randomised controlled trial

**DOI:** 10.1186/1471-2458-13-750

**Published:** 2013-08-13

**Authors:** Ruben A Kraaijeveld, Frederieke G Schaafsma, Cécile RL Boot, William S Shaw, Ute Bültmann, Johannes R Anema

**Affiliations:** 1 Department of Public and Occupational Health, EMGO+ Institute for Health and Care Research, VU University Medical Center, PO Box 7057, Amsterdam, 1007 MB, The Netherlands; 2 Research Centre for Insurance Medicine, Collaboration between AMC-UMCG-UWV-VUmc, Amsterdam, The Netherlands; 3 Body@Work, Research Center Physical Activity, Work and Health, TNO-VU University Medical Center, Amsterdam, The Netherlands; 4 Liberty Mutual Research Institute for Safety, Hopkinton, MA, USA; 5 Department of Health Sciences, Community & Occupational Medicine, University Medical Center Groningen, University of Groningen, Groningen, The Netherlands

**Keywords:** Participatory Approach, Sick leave, Supervisors, RCT

## Abstract

**Background:**

The burden of sick leave for society and organisations underlines the urgent need to prevent sick leave. An effective workplace intervention for organisations to shorten sick leave episodes is the Participatory Approach (PA). In this study, we hypothesize that implementation of the PA for supervisors within organisations may prevent sick leave as well. However, implementation of the PA within an organisation is difficult, and barriers at different levels (employee, supervisor and organisational) exist. Therefore, the primary aim of this study is to evaluate the effectiveness of a multifaceted implementation strategy of the PA.

**Methods:**

In a cluster randomised controlled trial (RCT) a multifaceted implementation of the PA will be compared with a minimal implementation strategy of the PA. Participating organisations are a university medical centre, a university and a steel factory. Randomisation will take place at department level. Intervention departments will receive a multifaceted implementation strategy of the PA, which incorporates a working group, supervisor training, and supervisor coaching. Control departments will receive the minimal implementation strategy of the PA, consisting of written information only. The primary outcome measure is self-efficacy of supervisors in joint problem solving to improve work functioning of employees with health complaints and to prevent sick leave. A secondary outcome measure at supervisor level is self-efficacy in communicating with employees about situations of reduced work functioning or being at risk for sick leave. Secondary outcome measures at employee level are attitude, self-efficacy, and social influence, with regard to addressing situations of reduced work functioning or being at risk for sick leave, as well as work functioning, psychological well being, and sick leave. Measurements will take place at baseline, and after six and twelve months follow-up. A process evaluation will be performed as well.

**Discussion:**

This study will be relevant for all organisations with employees at risk for sick leave in health care, education, and industry. Study results will give an insight into the effectiveness of the multifaceted implementation strategy of the PA for supervisors to improve work functioning of employees with health complaints, and to prevent sick leave.

**Trial registration:**

NTR3733

## Background

Sick leave is still a major burden for societies in the Netherlands and other western industrialised countries [1-4]. Besides personal suffering, sick leave also entails extra efforts and costs for organisations and their employees. Human resources (HR) or occupational health services have to take care of sick-listed employees; work remains to be done; colleagues have to take over work; and supervisors may have to support their sick-listed employees. In the Netherlands, the average absence rate was 4.2 percent in 2011, which means a loss of 9 working days per employee per year [3,5]. In addition, direct costs of sick leave for Dutch society in 2010 are estimated at more than €12 billion [2]. By comparison, sick leave costs in the UK in 2013 are estimated at ₤29 billion and in Australia in 2010 at A$30 billion [1,3,4]. This burden of sick leave underlines the urgent need to prevent sick leave.

An effective workplace intervention for organisations to shorten sick leave episodes is the Participatory Approach (PA) [6-9]. PA is a stepwise workplace intervention protocol for supervisors and employees to identify and jointly solve barriers for return to work (RTW). A study by Lambeek et al. demonstrated that an intervention programme with PA for employees sick-listed due to low back pain resulted in a decrease of 120 days to sustainable RTW [8]. Another study also reported that PA is effective for sustainable RTW for employees sick-listed due to distress, if they had the intention to return to work despite symptoms [9]. Although PA is proven to be an effective intervention, it is still not common practice in Dutch organisations [10]. Therefore, an effective strategy for the implementation of PA within organisations may need more research.

Besides applying PA to shorten sick leave episodes, we hypothesize that PA can be used for the prevention of sick leave as well. Although in previous studies PA is applied by an occupational health professional (OHP), for the prevention of sick leave we argue that supervisors, with adequate training and coaching by an OHP, are also able to apply PA. Moreover, supervisors are the first people who can identify employees with reduced work functioning or who are at risk for sick leave due to health complaints. Several studies have already demonstrated the influence of supervisors’ behaviour on work functioning and well-being of employees [11-15]. Hence, we hypothesize that PA applied by supervisors will be effective in improving the work functioning of employees with health complaints and in preventing sick leave.

Regarding the implementation of the PA within an organisation, with reference to the prevention of sick leave, different barriers at organisational, supervisor, employee, and OHP levels exist [10,16,17]. First, the PA does not always correspond well with sick leave policies of organisations. For example, HR may not facilitate modified work for employees with work functioning problems [10,16]. Second, supervisors may experience a lack of self-efficacy in addressing work functioning problems of employees and in jointly realising solutions to these problems [10,16]. Third, supervisors may assume that employees at risk for sick leave – in particular, due to mental health problems – only need rest to recover for functioning at work [10]. Fourth, employees may experience a lack of support, empathy, or the possibility of joint problem solving with their supervisors. They may also encounter insufficient support from HR and organisational policies to recognise and solve work functioning problems [10]. Last, OHPs indicate insufficient knowledge and communication skills of supervisors regarding support for employees at risk for sick leave, or supervisors’ insufficient knowledge as to when to consult an OHP [10,16].

Because of these barriers at different levels, implementation of the PA requires a multifaceted strategy. Shaw et al. showed that working groups combined with training and coaching of supervisors is effective in the prevention of sick leave [17]. This study showed an increase of supervisors’ self-efficacy in their communication with employees with health complaints, a 47% decrease of sick leave episodes, and an 18% decrease of sick leave duration. In general, previous research on implementation of interventions within organisations showed that a multifaceted strategy has proved to be more effective than a single strategy [18]. Moreover, to realise changes within organisations, comprehensive strategies targeted at all different organisational levels are needed [18].

Consequently, the implementation strategy of this study incorporates three components: (1) a working group with representatives of managers, supervisors, employees, HR professionals, and OHPs, (2) supervisor training in PA by in-company OHPs, and (3) coaching of supervisors in PA by in-company OHPs. The main objective of this study is to evaluate the effectiveness of the multifaceted implementation strategy of the PA to increase supervisors’ self-efficacy in joint problem solving for the improvement of work functioning of employees with health complaints, and for the prevention of sick leave. Secondary objectives are: (1) to evaluate the effectiveness of this strategy to improve experienced support and work functioning of employees, (2) to prevent sick leave, and (3) to asses facilitators and barriers for the multifaceted implementation strategy of the PA at all organisational levels.

## Methods

To describe the methods of this study, the CONSORT statement was used [19].

### Study context

In the Netherlands, supervisors are expected to play an important role in the coordination of sick leave of employees and consequently in the prevention of sick leave. The Dutch Gatekeeper Improvement Act directs that employer and employee are both responsible for return to work (RTW). An employer is obliged to start RTW as soon as possible, for example, by offering (other) suitable work, and an employee is obliged to play an active role in the RTW process, for example, accepting other work activities. For this reason most Dutch organisations assign their supervisors as the first point of contact for employees in cases of reduced work functioning or sick leave. After more than six weeks of sick leave supervisor and employee jointly have to draw up a plan of action for RTW. The supervisor also has to manage an RTW file in which all actions and agreements regarding disability and RTW are recorded.

### Study design

This study is a cluster-randomised controlled trial (RCT) in which two implementation strategies will be compared. The PA will be implemented within three organisations: a steel factory, a university medical centre, and a university. Departments of these organisations will be allocated to either the intervention or control group. Intervention departments will receive the multifaceted strategy of the PA and control departments the minimal implementation strategy of the PA. Measurements will take place at baseline and after six and twelve months follow-up. The study protocol has been approved by the Medical Ethics Committee of the VU University Medical Center, Amsterdam, the Netherlands.

### Study population

Supervisors and their employees will be recruited from all three aforementioned organisations. These organisations have about 20,000 employees of which 1,400 have a supervisory role for 10 employees or more. Only departments with participating supervisors will be included in this study. Participation of both supervisors and their employees is voluntary.

#### Inclusion criteria

Eligible supervisors have a minimum age of 18 years, work at least 24 hours per week, and supervise at least 10 employees. Eligible employees have a minimum age of 18 years and work at least 24 hours per week.

#### Exclusion criteria

Supervisors and employees whose contracts will end within one year from baseline or who are not able to fill out questionnaires in the Dutch language will be excluded.

### Randomisation, stratification and blinding

Randomisation will be performed at department level to limit contamination between supervisors of the intervention and control groups. Departments within organisations will be matched as pairs, based on the number of participating supervisors and sick leave frequencies of departments. Randomisation of departments of each pair will be performed by an independent researcher. Inherent in the multifaceted implementation strategy of the PA, it is impossible to blind researchers, supervisors, managers, HR professionals, and OHPs.

### Multifaceted implementation strategy

Intervention departments will receive the multifaceted implementation strategy of the PA, which incorporates three components. After baseline measurement (month 1), a working group (month 2), supervisor training (month 3) and supervisor coaching (months 4–12) will be carried out.

#### First component: working group

A two-hour working group meeting, chaired by an in-company OHP, will be organised before, and nine months after, supervisor training. The working group consists of a maximum ten representatives of managers, supervisors, employees, HR professionals, and OHPs. In the first meeting, baseline results will be presented to elaborate on signals of reduced work functioning, situations in which supervisors should apply PA, and barriers to and facilitators for implementation of PA. Working group results will be summarised in a manual for supervisor training and coaching. In a follow-up meeting, the working group will be asked to evaluate barriers and facilitators for implementation of PA.

#### Second component: supervisor training

Supervisors will be trained in applying PA by in-company OHPs in a four-hour session and an optional follow-up meeting of two hours. Before supervisor training, in-company OHPs are trained by the research team (RAK, FGS, CRLB, JRA) and provided with a training manual. For this study, application of PA is adjusted for the prevention of sick leave and worded in a workplace intervention protocol for supervisors. This protocol consists of seven steps to identify and solve employees’ work functioning problems due to health complaints (see Table 1). The protocol incorporates three supervisor-employee meetings with the supervisor both as participant and process leader. In the first meeting the employee is informed by the supervisor about the protocol and about both their roles in the next meetings. If needed, an employee may ask for an in-company OHP to act as a neutral process leader when applying this protocol.

**Table 1:** Workplace intervention protocol for supervisors


**Meeting 1**	Step 1. Supervisor addresses employee’s reduced work functioning due to health complaints or being at risk for sick leave. Supervisor informs employee about the workplace intervention protocol of the study.
**Preparation**	Step 2. Employee makes an inventory of work tasks and activities, and prioritises work functioning problems within activities. Employee thinks of possible solutions for the two most important work functioning problems.
	Step 3. Supervisor makes an inventory of work tasks and activities for the employee, and prioritises work functioning problems within activities. Supervisor thinks of possible solutions for the two most important work functioning problems.
**Meeting 2**	Step 4. Supervisor and employee discuss work functioning problems and associated solutions, and assess the applicability of these solutions.
	Step 5. Supervisor and employee agree on action plan to realise solutions. If they don’t agree, supervisor asks in-company occupational health professional to act as process leader.
**Realisation**	Step 6. Solutions are prepared and realised.
**Meeting 3**	Step 7. Supervisor and employee evaluate action plan and realised solutions.

#### Third component: supervisor coaching

As a follow-up of the training, supervisors will be coached by an in-company OHP during the application of PA, if they want to. For example, a supervisor can prepare an up-coming meeting with an employee together with the in-company OHP. In addition, if supervisors find it difficult to be a participant as well as a process leader, they can ask an in-company OHP to act as process leader.

### Minimal implementation strategy

Control departments and their supervisors and employees will receive the minimal implementation strategy, consisting of distribution of written information about PA.

### Use of co-interventions

During this study, department managers and supervisors will be regularly asked about ongoing or planned reorganisations and in-company projects, which may influence results of this study.

### Outcome measures

#### Primary outcome measure

The primary outcome measure is self-efficacy of supervisors in joint problem solving to improve work functioning of employees with health complaints, and to prevent sick leave. Three items of the competence scale of the Empowerment questionnaire of Spreitzer et al. are modified to the context of this study [20]. For example, ‘I am confident about my ability to find and realise solutions together with my employee’. In accordance with the scale of Spreitzer et al. a seven-point Likert scale is used, ranging from 1 (totally disagree) to 7 (totally agree) [20].

#### Secondary outcome measures

Secondary outcomes will be measured both at supervisor and employee level.

#### Supervisors

Self-efficacy in communicating with employees about situations of reduced work functioning or being at risk for sick leave will be measured with 3 self-formulated items; for example, ‘I am self assured about addressing these situations together with my employee’. Also supervisors’ attitude, social influence and intention to address situations of reduced work functioning or being at risk for sick leave will be measured with self-formulated items. Three items are used to assess attitude; for example, ‘to address and solve these situations is important for me’. Two items are used to assess social influence; for example, ‘employees expect me to find and realise solutions together’. And one item is used to assess intention; for example, ‘it is very likely that my employee and I would find and realise solutions together’. Response categories for all items range from 1 (totally disagree) to 5 (totally agree).

#### Employees

Employees’ attitude, self-efficacy, and social influence about addressing situations of reduced work functioning or being at risk for sick leave will be measured by self-formulated items. Three items are used to assess attitude; for example, ‘in these situations it is important to inform your supervisor in time’. Three items will be used to assess self-efficacy; for example, ‘I have mastered the skills to address these situations with my supervisor’. Social influence will also be measured with three items; for example, ‘my organisation stimulates me to address these situations with my supervisor’. Response categories for all items range from 1 (totally disagree) to 5 (totally agree). Work functioning will be measured with ten items of a Dutch questionnaire about health and work [21]; for example, ‘I was at work but owing to health complaints I had to slow down my work pace’. Response categories are (almost) never, sometimes, often, (almost) always. Psychological well-being of employees will be obtained with the distress screener of the Four-Dimensional Symptom Questionnaire (4DSQ) of Terluin et al. [22], the UWES-9 [23], and the ‘need for recovery’ scale [24]. In addition, return-to-work self-efficacy of employees will be measured with the RTWSE-19 [25].

#### Sick leave

Sick leave frequency and duration (in calendar days) of employees per supervisor, from six months before baseline up to twelve months follow-up, will be measured with both self-reported and objective data. At all measurements, employees will be asked to report sick leave frequency and duration(s) within the last six months. Sick leave data will also be derived from databases of HR and contracted occupational health services.

#### Prognostic measures & possible confounders

At baseline and after twelve months, individual work-related factors of supervisors and employees such as department, job type, and working hours per week will be measured. Additionally, one single item, namely, ‘I am satisfied with my job’, will be used to assess job satisfaction of supervisors and employees. Response categories are never, rarely, sometimes, often, and always. General health of supervisors and employees will be assessed with one item of the SF-36 [26], namely, ‘In general, would you say your health is: excellent, very good, good, fair, or poor?’ Furthermore, three subscales of the Job Content Questionnaire (JCQ) will be used to assess decision authority, supervisor support, and job insecurity of employees [27]. Experienced leadership style by employees will be measured with the Global Transformational Leadership scale (GTL) [28]. At baseline, individual factors such as age, gender, and level of education of supervisors and employees will be obtained. Supervisors will also be asked if they are familiar with PA and if they already apply PA.

### Process evaluation

The framework of Linnan and Steckler will be used to evaluate the implementation process of the PA within the three organisations [29]. The components reach, dose delivered, dose received, fidelity, and recruitment will be assessed after six and twelve months. Data for the process evaluation will be gathered from supervisors and employees using questionnaires and via semi-structured interviews. In a follow-up meeting, representatives of the working group will be asked about barriers and facilitators for the implementation of the PA within their organisation.

### Sample size

The sample size calculation is based on a 10% hypothesized increase in self-efficacy of supervisors in joint problem solving to improve work functioning of employees with health complaints, as a result of the multifaceted implementation strategy of the PA. An intra-class correlation coefficient (ICC) of 0.05 is used to adjust for the cluster randomised design at department level. By using the ICC, the calculation formula for a 10% increase in self-efficacy with the mean score of 6.02 and a SD of 0.88 of the competence scale of Spreitzer et al. leads to a total sample size of 107 supervisors [20], assuming a dropout rate of 20% and taking into account a power (1-beta) of 0.80 and an alpha of 0.05.

### Data collection procedure

Data for outcome and prognostic measures, as well as possible confounders, will be gathered using online questionnaires for supervisors and their employees. For those who do not have regular computer access, a paper version of the questionnaire will be sent via postal mail.

### Statistical analyses

Outcome measures will be compared between both groups at baseline, six, and twelve months follow-up. Primary analyses will be performed at supervisor level and secondary analyses at supervisor and employee level. To assess the success of randomisation, descriptive statistics will be used comparing the baseline measurements of the two groups. In case of unsuccessful randomisation, there will be adjustments for baseline differences. The primary independent variable in the analyses will be binominal representing the allocation to either the intervention or control group. The primary dependent variable is self-efficacy of supervisors in joint problem solving to improve work functioning of employees with health complaints. Changes in primary and secondary outcome variables between baseline and follow-up measurements will be compared between the two groups using linear mixed models at supervisor and employee level. All statistical analyses will be performed according to the intention-to-treat principle and, in case of compliance differences of supervisors, per protocol analyses will be performed as well.

## Discussion

The present study will evaluate the effectiveness of the multifaceted implementation strategy of the PA, compared with a minimal implementation strategy of the PA, within three organisations. The multifaceted implementation strategy aims to tackle barriers for implementation of the PA at organisational, supervisor, employee, and OHP levels. Representatives of all stakeholders will be involved in a working group and they should identify specific barriers per organisation for implementation of the PA. The implementation strategy especially aims at the supervisor level, because several studies have demonstrated the influence of supervisors’ behaviour on work functioning and well-being of employees [12-15]. Moreover, qualitative studies report lacking of self efficacy and communication skills of supervisors to support employees with work functioning problems or who are at risk for sick leave as important barriers for implementation of the PA [10,16].

This study is the first that will evaluate the application of PA by supervisors to prevent sick leave. Earlier research showed that PA applied by OHPs is effective in shortening sick leave periods [8,9]. Furthermore, implementation of PA for the primary prevention of sick leave corresponds well to current sick leave policies within Dutch organisations. Applying PA by supervisors for the prevention of sick leave will stimulate efforts of both supervisors and employees in improving work functioning of employees with health complaints. Because in-company OHPs are trained by the research team, knowledge and skills about the application of PA are maintained within each organisation. Accordingly, this train-the-trainer principle enables sustainable implementation of PA within each organisation.

### Methodological considerations

This study has some important strengths. First, a cluster-RCT design will be used to evaluate the effectiveness of the multifaceted implementation strategy. Within implementation studies, RCTs are considered the gold standard in investigating the effectiveness of an intervention untainted by bias [7,19]. In contrast to other study designs, an RCT design will control for a priori differences (e.g. willingness of supervisors to participate) and unforeseen factors such as organisational changes, financial problems, and external influences. Second, by cluster randomisation of supervisors at department level, contamination of participating supervisors should be limited as much as possible. By assigning supervisors and their employees per department to either the intervention or the control group will make it less likely that participants of both groups will come into contact with each other.

A third strength of this study is that the PA will be implemented within three different types of organisations; hence, the generalisability of the study results is expected to be good. Supervisors and their employees will be recruited from departments of a steel factory, a university medical centre, and a university. Therefore, the total population of supervisors and their employees will include various types of jobs and professions. Although sick leave causes are expected to differ within these three organisations, supervisors will be able to apply PA for employees with work functioning problems or who are at risk for sick leave, independently of the health related cause. PA can be applied for all types of health complaints, as the intervention focuses on improving work functioning despite health complaints [8,9].

There are also some limitations. First, recruitment bias may influence results at both supervisor and employee level. Because participation of the supervisor is voluntary, participating supervisors may already be more supportive or interested in supporting employees at risk for sick leave, which could lead to ceiling effects. Supervisors who are not motivated to support employees at risk for sick leave may also be less skilled in supporting. Accordingly, employees who have a bad working relationship with their supervisor or do not want to discuss reduced work functioning due to health complaints will probably be underrepresented. Second, inherent in the multifaceted implementation strategy, it will be impossible to blind researchers, in-company professionals, managers and supervisors for the allocated implementation strategy. In addition, because working groups will take place at organisational level, it is possible that control departments have some benefit from working group results.

Third, data of most outcome measures of this study is self-reported and may be influenced by information or recall bias. For example, it is reasonable that supervisors of the intervention departments will fill out questionnaires at follow-up more accurately than those of the control group. Training and coaching of supervisors will probably create more awareness for questionnaire topics and items. In addition, supervisors in the control group will perhaps be less motivated to fill out questionnaires at follow-up after being allocated to the control group.

### Relevance/impact of results

This study will be relevant for all organisations with employees at risk for sick leave in health care, education, and industry. PA is an effective workplace intervention to shorten sick leave periods. However, how to implement this intervention within an organisation effectively, and whether PA is also effective for the prevention of sick leave is still unclear. This study will give insight into the effectiveness of the multifaceted implementation strategy of the PA for supervisors, within three different types of organisations, to increase supervisors’ self-efficacy in supporting employees at risk for sick leave. Results of the study will become available in 2014.

## Competing interests

The authors declare that they have no competing interests.

## Authors’ contributions

RAK wrote the initial protocol. FGS, CRLB, UB, WSS and JRA contributed to the further writing of the manuscript. All authors read and approved the final version of the manuscript.

## Pre-publication history

The pre-publication history for this paper can be accessed here:

http://www.biomedcentral.com/1471-2458/13/750/prepub
